# The detective, prognostic, and predictive value of DNA methylation in human esophageal squamous cell carcinoma

**DOI:** 10.1186/s13148-016-0210-9

**Published:** 2016-04-22

**Authors:** Kai Ma, Baoping Cao, Mingzhou Guo

**Affiliations:** Department of Thoracic Surgery, the Affiliated Hospital of Qingdao University, Qingdao, China; Department of Gastroenterology and Hepatology, Chinese PLA General Hospital, #28 Fuxing Road, Beijing, 100853 China

**Keywords:** DNA methylation, Esophageal cancer, Epigenome, CHFR, Wnt signaling, DNA damage repair

## Abstract

Esophageal cancer is one of the most common malignancies in the world. Squamous cell carcinoma accounts for approximately 90 % of esophageal cancer cases. Genetic and epigenetic changes have been found to accumulate during the development of various cancers, including esophageal squamous carcinoma (ESCC). Tobacco smoking and alcohol consumption are two major risk factors for ESCC, and both tobacco and alcohol were found to induce methylation changes in ESCC. Growing evidence demonstrates that aberrant epigenetic changes play important roles in the multiple-step processes of carcinogenesis and tumor progression. DNA methylation may occur in the key components of cancer-related signaling pathways. Aberrant DNA methylation affects genes involved in cell cycle, DNA damage repair, Wnt, TGF-β, and NF-κB signaling pathways, including *P16*, *MGMT*, *SFRP2*, *DACH1*, and *ZNF382*. Certain genes methylated in precursor lesions of the esophagus demonstrate that DNA methylation may serve as esophageal cancer early detection marker, such as methylation of *HIN1*, *TFPI-2*, *DACH1*, and *SOX17. CHFR* methylation is a late stage event in ESCC and is a sensitive marker for taxanes in human ESCC. *FHIT* methylation is associated with poor prognosis in ESCC. Aberrant DNA methylation changes may serve as diagnostic, prognostic, and chemo-sensitive markers. Characterization of the DNA methylome in ESCC will help to better understand its mechanisms and develop improved therapies.

## Background

Esophageal carcinoma is the sixth leading cause of cancer-related mortality and the eighth most common cancer worldwide [1]. Esophageal cancer has two main subtypes-esophageal squamous cell carcinoma (ESCC) and esophageal adenocarcinoma (EAC). ESCC is the predominant histological type and accounts for 90 % of the cases of esophageal carcinoma worldwide [2]. Tobacco smoking and alcohol consumption are two major risk factors in ESCC [3–5], while gastroesophageal reflux disease (GERD) [6], obesity, and diet [7] were recognized as risk factors for EAC. Despite surgery or chemo-radiotherapy, the prognosis of esophageal cancerstill remains poor with the overall 5-year survival ranging from 15 to 25 % [2, 8, 9]. The mechanisms involved in ESCC remain unclear. Therefore, a clearer understanding of esophageal cancer and subsequent treatment advances are in urgent need.

Both aberrant genetic and epigenetic changes have been demonstrated to contribute to human ESCC initiation and progression [10–12]. This review focuses on recent advances involving DNA methylation and its clinical application in human ESCC.

### Epigenetic alterations induced by risk factors of ESCC

As mentioned above, nutrition and the consumption of tobacco and alcohol contribute to ESCC carcinogenesis. Talukdar et al. found that promoter region hypermethylation is associated with tobacco consumption by analyzing a group of tumor suppressor genes in human ESCC [13]. Similar results were obtained from another group [14]. Tobacco contains 4-(methylnitro-samino)-1-(-3-pyridyl)-1-butanone (also known as nicotine-derived nitrosamine ketone (NNK)) and Benzo[α]pyrene, which were found to modulate DNA methylation. NNK induced hypermethylation of multiple tumor suppressor genes in liver and lung tumors of rat and mouse models [15–17]. Another study found that NNK attenuated DNMT1 degradation and also induced its nuclear accumulation resulting in subsequent hypermethylation of promoters of tumor suppressor genes in animal and human lung cancer [18]. Benzo[α]pyrene diol epoxide (BPDE), a carcinogen present in tobacco smoke and environmental pollution, has been shown to induce gene mutations (such as in *P53* and *KRAS* genes) in vitro [19–22]. A recent study demonstrated that BPDE induced *RARβ2* promoter region methylation by recruiting DNA (cytosine-5-)-methyltransferase 3 alpha (DNMT3A) to its promoter region [23]. Another group identified that promoter methylation of the fragile histidine triad (*FHIT*) gene in ESCC was significantly associated with exposure to tobacco smoke [24]. Methylation frequency of the mutS homolog 3 (*MSH3*) gene promoter was significantly higher in tumor samples from smokers compared to tumor samples from non-smokers [25]. *P16* methylation is associated with occupational airborne dust exposures. The methylation rate of *p16* is 8.7 times higher in patients that have been exposed to occupational airborne dust compared to patients without such exposure [26].

Many epidemiological studies have consistently shown that alcohol consumption is an etiological factor of human ESCC. ESCC has a stronger association with alcohol consumption than cancers of other organ sites [27, 28]. Genetic polymorphisms of ethanol-metabolizing genes, such as acetaldehyde dehydrogenase (ALDH) and alcohol dehydrogenases (ADH), are associated with ESCC [29–31]. Tobacco use and alcohol drinking have synergistic effects on carcinogenesis, where combined use explained more than 61 % of ESCC [32, 33]. In the liver, ethanol is oxidized to acetaldehyde by ADH [34, 35]. Chronic alcoholism increases the requirements for methyl groups and causes dietary methyl group deficiency [36]. Deficiency of S-adenosylmethionine, folate, and betaine due to destruction by acetaldehyde is common in alcoholics. Inhibition of methyl group transfer regulates the expression of genes involved in carcinogenesis [37, 38]. Several studies have shown that alcohol is associated with global DNA hypomethylation and tumor suppressor gene promoter region hypermethylation in human esophageal, hepatic, and colorectal cancers [39].

### Aberrant DNA methylation changes in human ESCC

Global genomic DNA hypomethylation and promoter region hypermethylation have been extensively studied in human cancers, including ESCC [10, 11, 40–43]. Aberrant DNA methylation is involved in the major components of cell cycle, DNA damage repair, and cancer-related signaling pathways.

#### Cell cycle-related genes

P14, p15, and p16 are cyclin-dependent kinase (CDK) inhibitors that negatively regulate the G1-S transition in the cell cycle. *P16* is frequently methylated in ESCC, while methylation of *p14* and *p15* is relatively infrequent in ESCC [44]. *P16* is methylated in precursor lesions of the esophagus. This suggests that P16 is involved in the early stages of esophageal carcinogenesis.

The RAS association domain family 1A (RASSF1A) is a microtubule-binding and stabilizing protein. RASSF1A interacts with microtubules and inhibits M-phase cell progression [45]. *RASSF1A* is frequently methylated in ESCC [46–48]. RASSF10 is a new member of the Ras-association family. RASSF10 inhibits cell proliferation and induces G2/M phase arrest. *RASSF10* is methylated in 44.3 % of ESCC [49]. Checkpoint with FHA and ring finger (CHFR) is another protein involved in mitotic checkpoint regulation [50]. CHFR induced G2/M phase arrest in ESCC. *CHFR* is frequently methylated in various cancers [51–54]. *CHFR* is methylated in 45 % of human invasive ESCC and infrequently methylated in esophageal early lesions, suggesting that *CHFR* methylation may serve as a late stage marker in ESCC. Methylation of *CHFR* sensitized ESCC cells to taxanes [54].

#### DNA repair genes

Fragile histidine triad (FHIT) is regarded as a “caretaker,” and loss of this caretaker function initiates the onset of genome instability and cancer development [55]. In some tumors that are associated with environmental carcinogens, alterations in the *FHIT* gene occur quite early in the development of cancer [56]. *FHIT* is frequently methylated in the early stages of ESCC, and aberrant methylation of *FHIT* is associated with poor prognosis and tobacco exposure [24].

The mismatch repair (MMR) system recognizes base–base mismatches and insertion or deletion loops (IDLs) in double-stranded DNA to degrade the newly synthesized error region and re-synthesize the correct second strand according to the template [57]. The human MMR system includes *MLH1*, *MLH3*, *MSH2*, *MSH6*, *PMS1*, and *PMS2* genes. Defective MMR increases mutation rates up to 1000-fold and leads to microsatellite instability (MSI) to result in carcinogenesis [58]. Germline MMR mutation gives rise to hereditary nonpolyposis colorectal cancer (CRC) accounts for ~3 % of all CRCs. Human MMR deficiency is mainly happened to *MLH1* and *MSH2* genes. By contrast with HNPCC, sporadic cancers are rarely found to have mutations in the *MLH1* or *MSH2* genes. In the population-based studies, the prevalence of MSI among CRCs is approximately 15 %. Mismatch repair deficiency can be inherited mutations or biallelic *MLH1* promoter region hypermethylation [59]. Methylation may be served as “second hit” for carcinogenesis. *MLH1* is frequently methylated in sporadic CRC and other tumors, while *MSH2* was not methylated in any of the sporadic CRCs [60–62]. In human esophageal squamous cell carcinoma, *MLH1* and *MSH2* are methylated in 33–62 % and 29–32 % of cases, irrespectively [40, 44, 63]. The expression of MLH1 and MSH2 were silenced by promoter region hypermethylation [44, 64, 65].

O6-methylguanine-DNA methyltransferase (MGMT) is a DNA damage repair enzyme that protects cells from G to A mutations by removing methyl or alkyl groups from guanine after chemical modification [66]. *MGMT* is frequently methylated in human ESCC, and methylation of *MGMT* sensitized ESCC to temozolomide treatment [44, 67].

#### Wnt signaling pathway genes

The canonical Wnt signaling pathway is involved in many biological processes, including embryogenesis and carcinogenesis [68, 69]. The activated Wnt/β-catenin signaling pathway may induce MYC, cyclin D1, and expression of other downstream genes, promoting cell proliferation, and leading to carcinogenesis. Numerous Wnt signaling components, including Wnt, secreted frizzled-related proteins (SFRPs), and β-catenin, are also of pivotal importance in the activation/inhibition of Wnt signaling.

Wnt signaling plays an important role in esophageal cancer initiation and progression [70]. Epigenetic regulation of key genes in the Wnt signaling pathway has been reported by several groups. *SFRP1* is methylated in 95 % of ESCC. *SFRP2* is methylated in 83 % of ESCC, and the expression of SFRP2 is regulated by promoter region hypermethylation [71]. SRY-box containing gene 17 (SOX17) is reported to play critical roles in the regulation of development and stem/precursor cell function through repression of Wnt pathway activity [72]. *SOX17* is frequently methylated in ESCC, and methylation of *SOX17* activated Wnt signaling. *SOX17* methylation is an early detection marker and is related to patients’ history of alcohol use [73]. Reduced expression of SOX17 is related to poor prognosis in ESCC [74]. Wnt inhibitory factor-1 (WIF1), one of the most important Wnt antagonists, is frequently down-regulated by promoter region hypermethylation in various types of cancer [75, 76]. Wnt-5a antagonizes Wnt signaling by promoting GSK-3—independent β-catenin degradation. *Wnt-5a* is frequently methylated in ESCC [77]. Adenomatous polyposis coli (*APC*) is methylated more frequently in human adenocarcinoma than in human ESCC [44, 78]. The 2-year survival rate is reduced in *APC*-methylated patients [79].

#### TGF-β signaling pathway genes

In cancer, transforming growth factor-β (TGF-β) signaling regulates tumor initiation, progression, and metastasis through a diverse repertoire of tumor–cell-autonomous and host–tumor interactions [80, 81]. TGF-β is regarded to be both a tumor suppressor and an oncogene [82]. In human prostate cancer, overexpression of TGF-β enhanced angiogenesis around the tumor, which increased the metastasis of prostate cancer. On the other hand, gallbladder tumors secrete TGF-β, which inhibits angiogenesis and results in reduced tumor growth. TGF-β signaling acts as a tumor suppressor during breast carcinogenesis, while TGF-β promotes breast cancer metastasis in the later stages [83].

The role of TGF-β signaling in human ESCC has not been extensively studied. There are only a few reports on the epigenetic regulation of TGF-β signaling in ESCC. The human runt-related transcription factor 3 (RUNX3), an important component of the TGF-β signaling pathway, is deleted in a variety of human cancers, including ESCC. *RUNX3* is frequently methylated in human ESCC [84]. Human Dachshund homologue 1 (DACH1) is a major component of the retinal determination gene network. Loss of DACH1 expression was found in breast, prostate, lung, endometrial, colorectal and hepatocellular carcinoma. DACH1 expression was regulated by promoter region hypermethylation in esophageal cancer. The methylation frequency increased with the progression of esophageal carcinogenesis. *DACH1* methylation is associated with poor differentiation and late tumor stage. Both in vivo and in vitro studies have demonstrated that DACH1 suppresses human esophageal cancer growth by activating TGF-β signaling [85]. F-box protein 32 (FBXO32) is recently identified as a TGF-β/Smad target gene [86]. FBXO32 is methylated in 52.3 % of human ESCC and methylation of FBXO32 is associated with poor 5-year overall survival [87].

#### NF-κB signaling pathway genes

Nuclear factor-κB (NF-κB) is a nuclear transcription factor. It was named NF-κB because it was found bound to an enhancer element of the immunoglobulin kappa light chain gene in the nucleus of B cells [88, 89]. The Rel/NF-κB transcription factor family is composed of several structurally related proteins including five cellular proteins: c-Rel, Rel A, Rel B, p50/p105, and p52/p100 [90]. Activation of NF-κB promotes cancer cell proliferation, invasion, and metastasis [91]. Sustained activation of NF-κB contributes to malignant progression and therapeutic resistance in most major human cancers [91, 92]. NF-κB is involved in the process of carcinogenesis induced by infections and carcinogens (such as 7,12-dimethylbenz(a)anthracene (DMBA)) in various cancers, including esophageal cancer [93–95].

The mechanism of NF-κB in ESCC remains to be elucidated. It has been reported that inhibition of NF-κB can increase the chemo-sensitivity of esophageal cancer (EC) cells in vitro [96]. The NF-κB inhibitor Bay11-7082 had significant antitumor effects on ESCC xenografts in nude mice by promoting apoptosis and inhibiting proliferation and angiogenesis, as well as reducing the metastasis of ESCC cells to the lungs, without significant toxic effects [97]. There are a limited number of studies on the epigenetic regulation of NF-κB signaling. It has been demonstrated that the zinc finger transcription factor, ZNF382, an inhibitor of NF-κB, is frequently methylated in human ESCC [98].

### The application of DNA methylation markers in the clinic

The best studied epigenetic modification is promoter region hypermethylation in tumor suppressor genes. DNA methylation represents the epigenetic biomarker with the highest translational potential due to its stable nature and reliable detection technologies [99]. DNA methylation patterns of certain genes may serve as early detection, prognostic, and chemo-sensitive markers, as well as therapeutic targets.

#### DNA methylation as ESCC detection and prognostic markers

The epigenetic characteristics of ESCC are not well identified compared to other cancers, including esophageal adenocarcinoma. As *LINE-1* elements constitute ~17 % of the human genome, the methylation status of *LINE-1* represents the global DNA methylation level [100]. *LINE-1* methylation has been shown to be highly variable among ESCC specimens, *LINE-1* hypomethylation is a marker of a poor prognosis in patients with early stage tumors, but not in those with advanced stage tumors [101]. Loss of imprinting (LOI) of insulin-like growth factor (*IGF2*) is associated with short time survival in ESCC [102]. The ten-eleven translocation (TET) family proteins can convert 5-methylcytosine (5-mC) to 5-hydroxymethylcytosine (5-hmC), which is now widely recognized as the “sixth base” in the mammalian genome, following 5-mC, the “fifth base” [103–108]. Loss of 5-hmC is a poor prognostic marker in kidney cancer [109]. The levels of 5-hmC are reduced in human ESCC, and the levels of 5-hmC are related to histologic grade [110].

Promoter region hypermethylation is found frequently in ESCC. DNA methylation changes were shown to have a progression tendency during esophageal carcinogenesis and progression, suggesting that DNA methylation is an early event in ESCC. Our previous studies found that *DAPK*, *p16*, *MGMT*, *MLH1*, *RARβ2*, *HIN1*, *TFPI-2*, *DACH1*, and *SOX17* were methylated in the precursor lesions of human esophageal epithelia [44, 73, 85, 111–113]. The methylation frequency increased with the progression of esophageal cancer. *CHFR* methylation is a late stage marker of ESCC [54]. Loss of CDH1 expression, a gene encoding E-cadherin, is related to tumor invasiveness, metastasis, and poor prognosis in ESCC. Methylation of *CDH1* was detected in 14–61 % of ESCC tumors, and it was associated with the recurrence of early stage [44, 114–117]. *RASSF1A* hypermethylation was significantly correlated with poorly differentiated tumors and advanced tumor stage [47, 118, 119]. *P16* methylation was associated with invasiveness and metastasis [120]. *FHIT* was methylated in the early stages of ESCC, and its methylation was associated with poor prognosis [24]. Our recent study found that *DACT2* methylation is frequently methylated in human esophageal squamous dysplasia and ESCC. *DACT2* methylation is associated with TNM stage and lymph node metastasis. These results suggest that *DACT2* methylation may serve as ESCC early detective and prognostic markers. *NKD2* is frequently methylated in human ESCC, and methylation of *NKD2* is associated with TNM stage and lymph node metastasis (data not shown). Additional methylation markers for ESCC are listed in Table 1. DNA methylation may serve as a marker for early detection, tumor recurrence, and prognosis in ESCC.

**Table 1:** Aberrantly methylated genes in ESCC

Gene	Histological type (M%)	Reference
HIN-1	LGD (31 %), MGD (33 %), HGD (44 %), ESCC (50 %)	[111]
DAB2	Dysplasia (67 %), ESCC (68 %)	[132]
PGP9.5	ESCC (42 %)	[133]
ECRG4	ESCC (69 %)	[134]
APC	ESCC (50 %), EAC (92 %)	[135]
FHIT	ESCC (33 %)	[24]
GNG7	ESCC (33 %)	[136]
CDH1	ESCC (43 %)	[137]
Integrin α4	ESCC (21 %)	[137]
Wif-1	ESCC (35 %)	[137]
MGMT	LGD (23 %), MGD (17 %), HGD (11 %), ESCC (33 %)	[44]
MLH1	LGD (8 %), MGD (17 %), HGD (33 %), ESCC (23 %)	[44]
RARβ2	LGD (13 %), MGD (33 %), HGD (44 %), ESCC (36 %)	[44]
TFPI-2	LGD (28 %), MGD (33 %), HGD (33 %), ESCC (67 %)	[112]
DACH1	LGD (19 %), MGD and HGD (42 %), ESCC (62 %)	[85]
SOX17	LGD (39 %), MGD and HGD (48 %), ESCC (65 %)	[73]
DAPK	LGD (28 %), MGD (25 %), HGD (11 %), ESCC (26 %)	[44]
P16	LGD (31 %), MGD (42 %), HGD (33 %), ESCC (52 %)	[44]
CHFR	LGD (2.9 %), MGD (0), HGD (12.5 %), ESCC (45 %)	[54]
RASSF10	ESCC (44.3 %)	[49]
ZNF331	ESCC (56.5 %)	[138]

*M%* methylation rate, *LGD* low-grade dysplasia, *MGD* middle-grade dysplasia, *HGD* high-grade dysplasia, *ESCC* esophageal squamous carcinoma

#### DNA methylation as a chemo-sensitive marker and therapeutic target in ESCC

Methylation patterns can be useful to assess clinical outcomes or response to chemotherapeutic agents. DNA methylation profiling has identified tumor-specific drug responsive markers in different cancers. The identification of biomarkers that predict response to chemotherapy is a component of precision medicine. For example, *MGMT* methylation was found to be associated with responsiveness to alkylator-based chemotherapy and an increase in overall survival and time to progression of gliomas [121]. In oxaliplatin-treated gastric cancer patients, overall survival was longer in the *MLH1* unmethylated group compared to the *MLH1* methylated group [60].

Many new epigenetic chemo-sensitive markers have been found in different cancer types [122]. Meanwhile, reports about DNA methylation patterns as chemo-sensitive markers in ESCC are very limited. CHFR is an early mitotic checkpoint gene that functions as a key player in controlling chromosomal integrity [123]. CHFR controls cell cycle progression at the G2/M checkpoint. Increased expression of CHFR leads to mitotic arrest. *CHFR* methylation is a sensitive marker for taxanes in human ESCC [54].

### Perspective

The landscapes of cancer genomes have already had an impact on the clinical care of cancer patients. The recognition that certain tumors contain activating mutations in driver genes encoding protein kinases has led to the development of small-molecule inhibitor drugs targeting those kinases [124]. However, the landscapes of the epigenome in all cancer types and their normal counterparts need to be completed. The Roadmap project for methylome mapping may generate more reference data sets for research and clinical use [125, 126].

Many DNA methylation markers have been reported for early detection, prognosis, therapeutic responsiveness, and therapeutic targets in different cancer types [127–130]. Targeting therapy based on aberrant genomic changes has already had an impact on the clinical care of cancer patients. While the value of epigenetic modifications in personalized medicines still not extensively studied. Epigenome-based personalized medicine may be suitable for human cancer patients with the recognition of cancer epigenome landscapes [131]. In ESCC, there are limited DNA methylation markers for early detection, prognosis, and chemo-responsiveness. We are far from having a full understanding of the molecular mechanisms responsible for the initiation and maintenance of the epigenetic abnormalities that help drive tumorigenesis. Therefore, we must continue to develop epigenetic biomarkers in ESCC.

## Conclusions

Epigenetic regulation of tumor suppressor gene expression plays an important role during esophageal carcinogenesis and progression. Aberrant DNA methylation patterns may serve as early detection, diagnostic, prognostic, and chemo-sensitive markers. While some important genes have already been identified to be frequently methylated in ESCC, mapping the landscape of the esophageal cancer epigenome has yet to be completed. Personalized therapy based on the ESCC epigenome will be developed in the future.

## Abbreviations

ADH: alcohol dehydrogenases
ALDH: acetaldehyde dehydrogenase
APC: adenomatous polyposis coli
BPDE: Benzo[α]pyrene diol epoxide
CDK: cyclin-dependent kinase
CHFR: Checkpoint with FHA and ring finger
CRC: colorectal cancer
DACH1: Dachshund homologue 1
DNMT3A: DNA (cytosine-5-)-methyltransferase 3 alpha
EAC: esophageal adenocarcinoma
ESCC: esophageal squamous carcinoma
FBXO32: F-box protein 32
FHIT: fragile histidine triad
GERD: gastroesophageal reflux disease
IDLs: insertion or deletion loops
MGMT: O6-methylguanine-DNA methyltransferase
MMR: mismatch repair
MSH3: mutS homolog 3
MSI: microsatellite instability
NNK: 4-(methylnitro-samino)-1-(-3-pyridyl)-1-butanone, also known as nicotine-derived nitrosamine ketone
RASSF1A: RAS association domain family 1A
RUNX3: human runt-related transcription factor 3
SFRPs: secreted frizzled-related proteins
SOX17: SRY-box containing gene 17
TGF-β: transforming growth factor-β
WIF1: Wnt inhibitory factor-1
